# Stent Selection for Endoscopic Ultrasound-Guided Drainage of Pancreatic Fluid Collections: A Multicenter Study in China

**DOI:** 10.1155/2014/193562

**Published:** 2014-06-11

**Authors:** Hui Lin, Xian-Bao Zhan, Si-Yu Sun, Xiu-Jiang Yang, Zhen-Dong Jin, Duo-Wu Zou, Zhao-Shen Li

**Affiliations:** ^1^Department of Gastroenterology, Shanghai Tenth People's Hospital, Tongji University School of Medicine, Shanghai 200072, China; ^2^Department of Gastroenterology, Changhai Hospital, Second Military Medical University, Shanghai 200433, China; ^3^Digestive Endoscopy Center, Sheng Jing Hospital of China Medical University, Shenyang 110004, China; ^4^Digestive Endoscopy Center, Fudan University Shanghai Cancer Center, Shanghai 200032, China

## Abstract

*Aims*. We attempted to establish some guidelines for the selection of transmural stents during endoscopic drainage of PFCs by retrospective review of the clinical data obtained from three tertiary hospitals. *Patients and Methods*. Clinical data of 93 patients with attempted endoscopic drainage of symptomatic PFCs were obtained through chart review and prospective follow-up. *Results*. Treatment success for acute pseudocyst (*n* = 67), chronic pseudocyst (*n* = 9), and WOPN (*n* = 17) was 95.3%, 100%, and 88.2%, respectively (*P* = 0.309). Clinical success for single-stent drainage was 93.9% (46/49) versus 97.4% (37/38) for multiple-stent drainage (*P* = 0.799). Secondary infection for single-stent drainage was 18.4% (9/49) versus 5.3% (2/38) for multiple-stent drainage (*P* = 0.134). Secondary infection for stent diameter less than or equal to 8.5 F was 3.4% (1/29) versus 17.2% (10/58) for stent diameter larger than or equal to 10 F (*P* = 0.138). * Conclusion*. EUS-guided transmural drainage is an effective therapy for PFCs. Single-stent transmural drainage of PFCs is enough and does not seem to influence clinical success. The number or diameter of stents does not seem to be associated with secondary infection.

## 1. Introduction

Pancreatic pseudocysts (PPs) and walled-off pancreatic necrosis (WOPN) are types of pancreatic fluid collections (PFCs) that arise as complications of acute pancreatitis or chronic pancreatitis, pancreatic trauma (including postsurgical), or neoplasia [1, 2]. The basis of the pancreatic injury is disruption of the pancreatic duct or side branches, resulting in the formation of a collection of fluids with or without solid debris. Inflammation is also an important factor. The indications for PFC drainage are driven symptom and/or the development of infection. Progressive enlargement of a fluid collection in asymptomatic patients is considered a reasonable indication for drainage [3, 4].

Endoscopic drainage of PFC can be achieved by transmural or transpapillary placement of endoprostheses or both [5, 6]. Although there are numerous reports of transmural drainage of PFC, experience was widely gained with compelling results [7–11]. There are also rare multicenter studies on the transmural stent selection associated with successful endoscopic ultrasound-guided drainage of PFC. Which outcome is better during endoscopic ultrasound- (EUS-) guided transmural drainage, single-stent placement or multiple-stent placement? Is secondary infection associated with the number or diameter of the stent? To answer these questions, the present study sought to establish some guidelines for the selection of transmural stents during EUS-guided drainage of PFCs by reviewing the long-term outcomes in patients with PFC endoscopic treatment over a 10-year period in three tertiary hospitals in China.

## 2. Patients and Methods

Data of all PFC patients who underwent EUS-guided drainage in three tertiary hospitals between November 2001 and December 2009 were entered into a computerized database. Patient records, including clinical notes, examination details, hospital course, and subsequent examinations or clinic visits, were reviewed. All the patients were followed up for more than two years through regularly scheduled clinical visits, telephone contacts, and/or contacts with the patients' family members to evaluate the long-term outcome.

All PFC patients who underwent EUS-guided transmural drainage were considered for inclusion. Exclusion criteria included patients who underwent examinations for diagnostic purposes only and those who were suspected as having neoplastic cysts. Indications for PFC drainage included abdominal pain, obstructive jaundice, infection, gastric outlet obstruction, early satiety, leakage, and progressive enlargement of fluid collection in asymptomatic patients.

### 2.1. Definitions

PFCs were classified as acute pseudocysts, chronic pseudocysts, or walled-off pancreatic WOPN according to Atlanta criteria and the previous literatures [12, 13]. An acute pseudocyst refers to a collection of pancreatic juices enclosed by a wall of nonepithelialized granulation tissue that requires at least 4 weeks to form, devoid of significant solid debris. A chronic pseudocyst refers to a collection of pancreatic juices enclosed by a wall of fibrous or granulation tissue that arises as a consequence of chronic pancreatitis. WOPN refers to a collection with good separation of devitalized (necrotic) tissue within a fluid-filled cavity and an associated fibrous wall lined by granulation tissue [14], as distinct from pancreatic necrosis that is not well defined and lacks a wall.

Technical success was defined as the ability to access and drain a PFC by placement of pancreatic-duct or transmural stents. Treatment success was defined as the complete resolution or decrease in size of the PFC to ≤2 cm on CT in association with clinical resolution of symptoms in a 6-week follow-up period. Complications were assessed at 24 h and at day 30.

### 2.2. Procedures

All patients underwent predrainage abdominal MRI or CECT to define the number, size, and location of each PFC, which were used to determine the accessibility and method of endoscopic drainage. Preprocedural broad-spectrum prophylactic antibiotics (levofloxacin) were administered in all patients.

EUS-guided transmural drainage was used for PFCs, which resulted in clear intraluminal bulging, or was in close proximity (<1 cm) to the gastrointestinal wall. Pancreaticography was performed after drainage whenever possible, especially in the patients with chronic pseudocysts. For communicating pseudocysts, an effort was made to place the catheter or stent across the area of leak or directly into the pseudocyst if possible. All drainage procedures were performed under general anesthesia. A linear echoendoscope was used to visualize pancreatic fluid collection. The pseudocyst was punctured with a needle under Doppler US guidance, and the cystic content was aspirated (if clinically appropriate) for fungal bacterial culture and chemical analysis. A 0.035-inch guidewire (Wilson-Cook) is then inserted through the needle into the fluid collection under fluoroscope guidance, the puncture site was dilated by a balloon catheter to 6–8 mm, and a double-pigtail transmural stent was inserted over the guidewire for drainage. Straight transmural stents were not used for drainage in any cases.

When multiple stents or an additional nasocystic catheter (NCC) was required, the “doublewire” approach was used, in which 2 or multiple guidewires were inserted through the same catheter before stent placement. The PFCs were followed by insertion of the second transmural stent or nasocystic catheter. The choice of initial drainage (via the nasocystic catheter or transmural stent or both) depended on the condition of the patients and the content of the collection.

Solid debris will not be evacuated completely by placement of transmural drainage stents alone. We used nasocystic catheters in combination with transmural stents in all sterile WOPN cases. The NCC is continually rinsed with 1 L saline solution for 24 h and manually flushed with 150 mL saline solution every 4 hours.

### 2.3. Statistics

Data are presented as mean ± standard deviation (SD) or median plus interquartile range (IQR), depending on distribution. Comparisons of the number of stents and the etiology, size, and location of the pseudocyst between groups were performed by the *t*-test for continuous data and the Kruskal-Wallis test, Mann-Whitney test, Pearson chi-squared test, or Fisher exact test for categorical data. Stepwise binary logistic regression was applied.

Commercial statistical software (SPSS for Windows, version 16.0; SPSS, Chicago, IL, USA) was used for data analysis. Two-tailed *P* values of less than 0.05 were considered statistically significant.

## 3. Results

EUS-guided transmural drainage was indwelled in 93 consecutive patients (60 men, 33 women; median age 49.0 ± 15.5 years, range 10–79 years). The etiology of the collections included acute pseudocyst (*n* = 67), chronic pseudocyst (*n* = 9), and WOPN (*n* = 17). The location of the collections was the pancreatic head in 30 cases or the body/tail in 63 cases. The median PFC diameter was 11.5 ± 4.9 cm (range: 3.5–25 cm).

The causes of the PFCs varied widely from gallstones in 50 (53.8%) cases, alcohol ingestion in 15 (16.1%) cases, idiopathic in 9 (9.7%) cases, trauma in 2 (2.2%) cases, hyperlipidemia in 2 (2.2%) cases, and others in 15 (16.1%) cases. The relative frequency of these causes in relation to the three types of PFCs is shown in Table 1. Abdominal pain was present in 37 cases, abdominal distension was present in 22 cases, cyst enlargement was present in 13 cases, fever was present in 9 cases, and seven patients were asymptomatic. Obvious extrinsic compression or bulging of the stomach or duodenal wall was present in 83 (89.2%) of the 93 PFC cases. Portal hypertension was present in 7 cases.

Of the 93 PFC patients, 93 patients underwent 106 episodes of endoscopic EUS-guided transmural drainage, including 90 (96.8%) patients whose drainage placement was technically successful and three patients whose drainage placement was failed due to bleeding from the puncture site, which was controlled by injection of 8 mL 1 : 10,000 epinephrine at the site of transmural puncture by using the needle-knife catheter.

The overall treatment success was 94.4% (85/90). Clinical success for bulging collection drainage was 95.1% (78/82) versus 87.5% (7/8) for nonbulging collection drainage (*P* = 0.379). Clinical success for transgastric drainage was 94.0% (79/84) versus 100% (6/6) for transduodenal drainage (*P* = 1.0). All seven patients with portal hypertension underwent endoscopic drainage successfully. Nasocystic catheters were placed in 18.9% of cases (17/90). Endoscopic drainage was successful in 15 of the 17 WOPN cases using combination of NCC and transmural stents (15/17, 88.2%).

In all patients with technical success, clinical success for single-stent drainage was 93.9% (46/49) versus 97.4% (37/38) for multiple-stent drainage (*P* = 0.799). The success rate for stent diameter less than or equal to 8.5 F was 96.5% (28/29) versus 94.8% (55/58) for stent diameter larger than or equal to 10 F (*P* = 1.00). The only patient who received 5 F transmural stenting developed fever three days after the procedure. Cyst fluid culture was positive for* Enterobacter cloacae* and* Candida albicans*, for which imipenem and fluconazole were administered for anti-infection therapy. The stent was removed 17 days later, and the patient was converted to open surgery because of failure of nasocystic drainage (Table 4).

The prognostic factors for the success of endoscopic drainage were evaluated (Tables 2 and 3). There was no significant difference in the success of alcoholic versus nonalcoholic pancreatitis (*P* = 0.580), gallstones versus nongallstones (*P* = 0.237), idiopathic versus nonidiopathic (*P* = 0.417), one pseudocyst versus multiple pseudocysts (*P* = 1.00), pseudocyst size less than or equal to 10 cm versus larger than 10 cm (*P* = 0.259), and location in the pancreatic head versus pancreatic body/tail (*P* = 0.871). Again, these were not statistically significant when age and gender were considered. Multivariate analysis showed that none of the variables tested was a significant predictor of success. In this retrospective study, the number of patients with portal hypertension, bulging, multiple pseudocysts, and cystduodenostomy was relatively small, which may affect the results of data analysis.

### 3.1. Complications

Complications occurred in 13 of the 90 patients (14.4%), including secondary infection (11/13), bleeding from the puncture site (1/13), and inadequate drainage (1/13). Complications were managed medically in 6 cases, endoscopically in 2 cases, and surgically in 5 cases. There was no procedure-related death.

Single-stent placement was performed in 49 patients, and secondary infection occurred in 9 of them. Multiple-stent placement was performed in 38 patients, and secondary infection occurred in 2 of them. There was no significant difference in secondary infection between the single-stent and multiple-stent placement groups (*P* = 0.134).

The stent diameter was less than or equal to 8.5 F in 29 patients, and only one patient developed secondary infection. The stent diameter was larger than or equal to 10 F in 58 patients, and 10 patients developed secondary infection. There was no significant difference in secondary infection when the size of double-pigtail stent diameter was considered (*P* = 0.138). Associations of secondary infection with different numbers or diameters of the stents are shown in Table 4.

### 3.2. Length of Hospital Stay

The mean length of hospital stay was 9.9 ± 10.1 days (range 1–50 days) for all patients undergoing transmural drainage. The median follow-up period of the 90 patients was 48 months (range 26–126 months). Pseudocysts recurred in 5 (5.6%) of the 90 patients, indicating that the overall endoscopic drainage of pseudocysts was successful.  Table 5 shows the outcomes after PFC endoscopic drainage.

## 4. Discussion

Endoscopic transmural drainage requests that the PFC is encapsulated and adjacent to the gastric or duodenal wall. The first series of transluminal endoscopic drainage was performed by blind puncture at the site of maximum impression on the gastric or duodenal wall. The presence of endoscopic bulging is prerequisite. The most common site of transmural entry for drainage of PFCs appears to be the transgastric route [15]. In recent years, EUS-guided transmural drainage of symptomatic pancreatic fluid collections has increasingly been performed [16–18]. During the EUS-guided procedure, EUS is used for direct real-time visualization of the entry site, thus facilitating drainage in the absence of endoscopic bulging and avoiding interposed blood vessels through Doppler US. It was shown that EUS could be used to guide pseudocyst drainage in the context of patients with portal hypertension, thereby reducing the bleeding risk [19]. In our study, all the 7 patients with portal hypertension underwent endoscopic drainage successfully. The treatment success rate for acute pseudocyst, chronic pseudocyst, and WOPN was 95.3%, 100%, and 88.2%, respectively (*P* = 0.309), consistent with other studies [20, 21]. Results of EUS-guided drainage from large series are shown in Table 6 [22–24].

Our study describes the experience at three high-volume (>800 EUS per year) referral centers. The endoscopic drainage procedure was successfully completed in 90 of the 93 patients in our series. The overall clinical success rate was 94.4% in the 90 patients who underwent EUS-guided transmural drainage successfully. It is worthy of mentioning that there was a relatively high proportion of acute pancreatic pseudocyst in this patient group. The main cause of PFCs is gallstones in China, which is different from other studies [25]. As most acute peripancreatic fluid collections can be spontaneously reabsorbed within the first several weeks after onset of acute pancreatitis without need for endoscopic drainage, acute PFC was excluded in the present study.

WOPN, which was described as organized pancreatic necrosis in the early the literature, usually arises as a consequence of severe acute necrotizing pancreatitis. If contrast-enhanced CT (CECT) obtained at the onset of pancreatitis demonstrates significant pancreatic necrosis (>30%), PFC likely contains necrotic material. EUS or MRI may also demonstrate solid debris. Therefore, EUS and MRI are valuable complementary means to document the presence of solid debris within the collection [26]. WOPN may be sterile or infected. The diagnosis of infected WOPN can be suspected when extraluminal gas is present on CECT. However, infected WOPN was mostly requested surgical debridement [27]. In the present series, all acute PPs and chronic PPs were devoid of significant solid debris; all WOPN were present in solid debris and sterile. Endoscopic drainage was impossible in the PFCs present in septa in the cyst, which must be drained by surgery. Song et al. [28] reported that septa and mural nodules were found more frequently in cystic tumors than pseudocysts. Therefore, all enrolled PFCs were absent from septa in the cyst.

Selection of transmural stents has long been a concern for clinicians. If effective PFC drainage can be achieved by single-stent placement, there is no need for multiple-stent placement, because multiple-stent placement is more difficult, takes a longer time, and runs higher risk of inducing complications. Interestingly, there was no significant difference in the success rate between single-stent and multiple-stent placement in our series (*P* = 0.799). Therefore, single-stent drainage of PFCs is enough and does not influence clinical success. The purpose of multiple-stent drainage was to adequately clear solid debris. For mostly PFCs without significant solid debris, single-stent drainage is enough. For some WOPN cases, solid debris is relatively small and can flow through single-stent to be cleared. If solid necrotic debris is so large as not to be able to flow through single-stent, multiple-stent drainage could not remove such debris and endoscopic necrosectomy must be adopted [29]. But these WOPN cases are rare in our study.

The commonly used diameter of stents is 7 F, 8.5 F, or 10 F. There was no significant difference in the success of stent diameter less than or equal to 8.5 F versus larger than or equal to 10 F (*P* = 1.00). It was found in our study that the only patient who used single 5 F stent drainage developed serious infection. Another patient who used 10 F stent drainage developed serious bleeding and was converted to open surgery. In our series 7 F or 8.5 F stent can be used for those PFCs without significant solid debris; this may decrease the chance of bleeding. In addition, the size of the working channel of a therapeutic linear echoendoscope is 3.7 mm, and placing a 7 F or 8.5 F stent may be faster and easier.

Cahen et al. [9] reported that bleeding was caused by erosion of a straight endoprosthesis through the cyst wall into major vessels. Therefore, double-pigtail transmural stents were inserted into all PFCs for drainage in our series. Straight transmural stents were not used for drainage in any cases. In our study NCC was used almost in all sterile WOPN cases. The success rate for combination of NCC and transmural stents drainage was 88.2% (15/17). Nevertheless, this could not conclude that NCC irrigation is helpful for evacuating solid necrotic debris. Prospective studies that compare internal drainage alone and combined use of internal drainage and NCC drain are required for evaluating long-term outcomes.

Complications occurred in 13 cases (14.4%, 13/90) after endoscopic therapy, including secondary infection in 11 cases, bleeding from the puncture site in one case, and inadequate drainage in one case. Infectious complications are common after endoscopic drainage because of possible introduction of bacteria during the procedure and/or incomplete evacuation of solid debris. NCC irrigation is helpful for slow debridement of solid necrotic debris. This has been shown to reduce the rate of superinfection [30].

In our study, secondary infection was a major complication (11/13). It is not known whether the number or diameter of the stents influenced the infection rate of PFC endoscopic drainage. Up to now, there are no generally accepted guidelines for selection of the stent size or number. Some researchers believe that multiple stents or large diameter stents are helpful for fluid collection and solid debris evacuation, but they also increase the back flow of bacteria from the gastric cavity, causing secondary infection. Interestingly, there was no significant difference in secondary infection between the single-stent and multiple-stent groups in our series (*P* = 0.134). There was no significant difference in secondary infection when the size of double-pigtail stent diameter was considered (*P* = 0.138). Therefore, selection of the stent size and number depends on adequate drainage. Use of prophylactic antibiotics 3 days before operation and one or two weeks after operation can prevent secondary infection [31].

In conclusion, this retrospective multicenter systematic study provides reliable estimates of the efficacy and transmural stent selection associated with successful EUS-guided drainage of pancreatic fluid collections. Single transmural stent drainage of PFCs is enough and does not seem to significantly influence clinical success. Secondary infection is the major complication in our study, but interestingly we find that the number or diameter of the stents does not seem to influence the infection rate of PFC endoscopic drainage.

## Figures and Tables

**Table 1:** Characteristics of patients with pancreatic fluid collections.

	Acute PP	Chronic PP	WOPN	Total
	(*n* = 67)	(*n* = 9)	(*n* = 17)	(*n* = 93)
Gender, men, *n* (%)	47 (50.5)	5 (5.4)	8 (8.6)	60 (64.5)
Age, median (IQR), y	47 (10–73)	56 (16–77)	53 (32–79)	49 (10–79)
Etiology, *n*				
Gallstones	36	5	9	50
Alcohol	10	0	5	15
Idiopathic	5	1	2	9
Hyperlipidemia	2	0	0	2
Trauma	2	0	0	2
Others	11	3	1	15
Location, *n*				
Pancreatic head	24	2	4	30
Pancreatic body/tail	43	7	13	63
Symptom, *n*				
Abdominal pain	28	4	5	37
Abdominal distension	14	3	5	22
Enlarging cyst	10	1	2	13
Fever	4	1	4	9
Asymptomatic	6	0	1	7
Others	5	0	0	5
Median diameter (cm)	11.5 ± 5.0	10.2 ± 3.5	11.9 ± 5.2	11.5 ± 4.9

Acute PP: acute pseudocyst, chronic PP: chronic pseudocyst, and WOPN: walled-off pancreatic necrosis.

**Table 2:** The treatment success rate in PFCs with different characteristics.

Prognostic factors	Clinical success, *n*	Success rate	*P* values
Acute pseudocyst	61/64	95.3%	0.309
Chronic pseudocyst	9/9	100%	
WOPN	15/17	88.2	
Alcoholic	13/14	92.9%	0.580
Nonalcoholic	72/76	94.7%	
Gallstones	49/50	98%	0.237
Nongallstones	36/40	90%	
One pseudocyst	78/83	94%	1.00
Multiple pseudocysts	7/7	100%	
Size ≤ 10 cm	48/49	98.0%	0.259
Size > 10 cm	37/41	90.2%	
Pancreatic head	29/30	96.7%	0.871
Pancreatic body/tail	56/60	93.3%	
Bulging collections	7/8	87.5%	0.379
Nonbulging collections	78/82	95.1%	
Transgastric drainage	79/84	94.0%	1.00
Transduodenal drainage	6/6	100%	

**Table 3:** Prognostic factors logistic regression for successful endoscopic ultrasound-guided drainage of PFCs (follow-up: 48 months).

Prognostic factors	*B*	Wald	*P* values
Gender	−17.944	0.000	0.998
Age	−16.274	0.000	0.998
Etiology	0.625	1.701	0.192
Classification	−18.341	0.000	0.998
Location of PFC	−0.772	0.399	0.528
Number of PFCs	−17.265	0.000	0.999
Size of PFC	1.735	1.436	0.231
Bulging	0.510	0.115	0.734
Site of drainage	−18.082	0.000	0.999
Portal hypertension	−19.413	0.000	0.999
Number of stents	−0.034	0.001	0.970
Type of stent	−0.287	0.021	0.884

*B:* coefficient value, Wald: Wald chi-squared value.

**Table 4:** Clinical success and secondary infection in cases using different numbers or diameters of stents.

	Clinical success	*P* values	Secondary infection	*P* values
Number of stents				
Single stents	93.9% (46/49)	0.799	18.4% (9/49)	0.134
Multiple stents	97.4% (37/38)		5.3% (2/38)	
Diameter of stents				
≤8.5 F	96.5% (28/29)	1.00	3.4% (1/29)	0.138
≥10 F	94.8% (55/58)		17.2% (10/58)	

**Table 5:** Outcomes after endoscopic drainage of PFCs.

	Acute PP	Chronic PP	WOPN	Total
NCC irrigation, *n*	0	0	17	17
Stent, *n*	64	9	17	90
Hospital days	7.6 ± 5.7	10.7 ± 1.4	18.1 ± 1.6	9.9 ± 10.1
Technical success, *n* (%)	64/67 (95.5)	9/9 (100)	17/17 (100)	90/93 (96.8)
Clinical success, *n* (%)	61/64 (95.3)	9/9 (100)	15/17 (88.2)	85/90 (94.4)
Complications, *n* (%)	9/64 (14.1)	0	4/17(23.5)	13/90 (14.4)
Recurrence, *n* (%)	3/64 (4.7)	0	2/17 (11.8)	5/90 (5.6)

Acute PP: acute pseudocyst, chronic PP: chronic pseudocyst, WOPN: walled-off pancreatic necrosis, NCC: nasocystic catheter.

**Table 6:** Results of EUS-guided drainage from large series.

Study, y	Number of PFCs	% technical success	% treatment success	Complication rates
Hookey et al. [17], 2006	116	96	93	11.2%
Lopes et al. [22], 2007	62	100	94	Immediate 3%, delayed 18%
Varadarajulu et al. [23], 2008	60	95	93	0%
Varadarajulu et al. [18], 2011	211	NA	85.3	8.5%
Künzli et al. [24], 2013	108	97	84	20%

NA: data not available.
